# Selective control of *Rhipicephalus microplus* in a dairy cattle herd from different genetic groups

**DOI:** 10.1590/s1984-29612022062

**Published:** 2022-12-16

**Authors:** Mariana Fogale de Andrade, Rodrigo Giglioti, Gunta Gutmanis, Bianca Tainá Azevedo, Cristiane Fernandes de Carvalho Fiorin, Anibal Eugênio Vercesi, Luciana Morita Katiki, Cecília José Veríssimo

**Affiliations:** 1 Instituto de Zootecnia – IZ, Agência Paulista de Tecnologia dos Agronegócios – APTA, Nova Odessa, SP, Brasil

**Keywords:** Cattle, tick, alternative control, partial treatment of the herd, Gado, carrapato, controle alternativo, tratamento parcial do rebanho

## Abstract

Alternatives for *Rhipicephalus microplus* control are needed in the light of its resistance to acaricides. One of the ways to decrease the use of acaricides in a herd is selective control (SC). In the present study, SC was evaluated in a dairy herd consisting of different genetic groups: Holstein, Jersey, crossbreed and Girolando. Ticks were counted in the right anterior third region on around 90 cows, totaling nine evaluations at intervals of 21 days. Commercial pour-on acaricide was applied only when the infestation was greater than or equal to eight ticks larger than 4 mm in the anterior third region. Tick counts were transformed into log_10_ and analyzed using mixed models. There was significant difference among groups: Holstein had the highest averages of tick numbers, as expected, although 34.3% did not receive tick treatment. In the other groups, SC reduced the use of acaricides by 79.1% for crossbreed, 81.5% for Jersey and 94.9% for Girolando. The criterion used for applying the acaricide successfully kept the tick population under control. The great advantage of SC was savings to the system, without harming the animals, in addition to generate fewer residues in the animals and in the environment.

## Resumo

A resistência de *Rhipicephalus microplus* a carrapaticidas gera a necessidade de alternativas de controle. Uma das formas de diminuir o uso de carrapaticida em um rebanho é o controle seletivo do carrapato (CS). O presente estudo avaliou o CS em rebanho leiteiro, constituído de diferentes grupos genéticos: Holandês, Jersey, mestiço e Girolando. Carrapatos foram contados na região do terço anterior direito, totalizando nove avaliações em cerca de 90 vacas, a intervalos ao redor de 21 dias. Carrapaticida comercial *pour-on* foi aplicado somente quando a infestação fosse igual ou superior a oito carrapatos maiores que 4 mm na região anterior. Dados de contagens foram transformados em log_10_ e analisados por meio de modelos mistos. Houve diferença significativa entre os grupos genéticos: vacas Holandesas apresentaram maiores médias no número de carrapatos, como esperado, embora 34,3% não tenha recebido tratamento carrapaticida; nas demais, o CS proporcionou diminuição do uso de carrapaticidas de 79,1% para as mestiças, 81,5% para as Jersey e 94,9% para as Girolando. O critério utilizado para aplicar o carrapaticida manteve a população de carrapatos sob controle. A grande vantagem do CS foi a economia ao sistema, sem prejudicar os animais, com menos resíduos para animais e meio ambiente.

## Introduction

Throughout Brazil, the climatic conditions under which cattle-rearing takes place are favorable for survival of the tick *Rhipicephalus microplus* (Maia et al., 2014). The losses caused by this tick have been estimated at more than U$ 3 billion dollars per year (Grisi et al., 2014).

Cattle ticks are controlled mainly based on using chemicals that are capable of killing them or inhibiting their development. However, with constant use of these synthetic chemicals, resistant strains are a reality nowadays (Higa et al., 2015). Therefore, there is a need to study alternatives for tick control. One of these alternative means for control is selective control (SC), which consists of applying acaricide when the tick infestation level goes above a certain limit. This selective control method was first used with great success for controlling haemonchosis in sheep, through the Famacha® method (Molento, 2009), and it has also been used for controlling other parasites, such as the cattle tick (Molento et al., 2013) and the horn fly (Almeida et al., 2013). Selective control is based on the fact that the parasite-host relationship follows a negative binomial distribution (Maiorano et al., 2019), in which a small portion of the population has the highest infestation levels. Application of selective control method significantly reduced *R. microplus* populations in beef cattle and in crossbred dairy herds, according to Molento et al. (2013) and Signoretti et al. (2006), respectively.

Thus, the aim of the present study was to evaluate the impact of selective tick control in a dairy herd consisting of four genetic groups: Holstein, Jersey, crossbreed and Girolando, over a consecutive nine-month period, using tick counts, degree of infestation in the perineum and body condition of the animals.

## Material and Methods

### Animals and management

Animals within the dairy production system of the Instituto de Zootecnia, located in the municipality of Nova Odessa, state of São Paulo, Brazil, (22º42’ S, 47º18’ W; 570 m altitude) were evaluated between June 2018 and February 2019.

The production system used by this farm was semi-intensive. The herd was fed with silage and concentrate in troughs that were placed in paddocks with grass and rest areas on all day. This allowed natural infestation by *R. microplus* larvae. The dairy cows left the paddocks only two times a day for milking. The cows with the lowest productivity remained on pasture all the time and did not receive supplementation.

Approximately 90 cows (lactating and dry) were evaluated on nine occasions, with a total of 798 observations. The cows belonged to four different genetic groups: Holstein, Jersey, Girolando (½ and ¾ Gir) and crossbreed (¾ and ^7^/_8_ Holstein and/or Jersey x Gir). For ease of daily management and feeding, the animals were grouped: lactating cows were grouped according to production and breed, with Holstein cows separated from the crossbred and Jersey cows, except for the least productive group, in which all breeds were put together and were kept in a pasture solely for this group. The cows were inseminated during the first months of lactation, and as they dried out they were allocated to the maternity group, in which all breeds shared the paddock. All the paddocks of the farm were naturally infested with *R. microplus* larvae. The grasses in the paddocks were of the genera *Panicum* and/or *Cynodon.*

### Assessment of tick infestation

**Figure 1:**
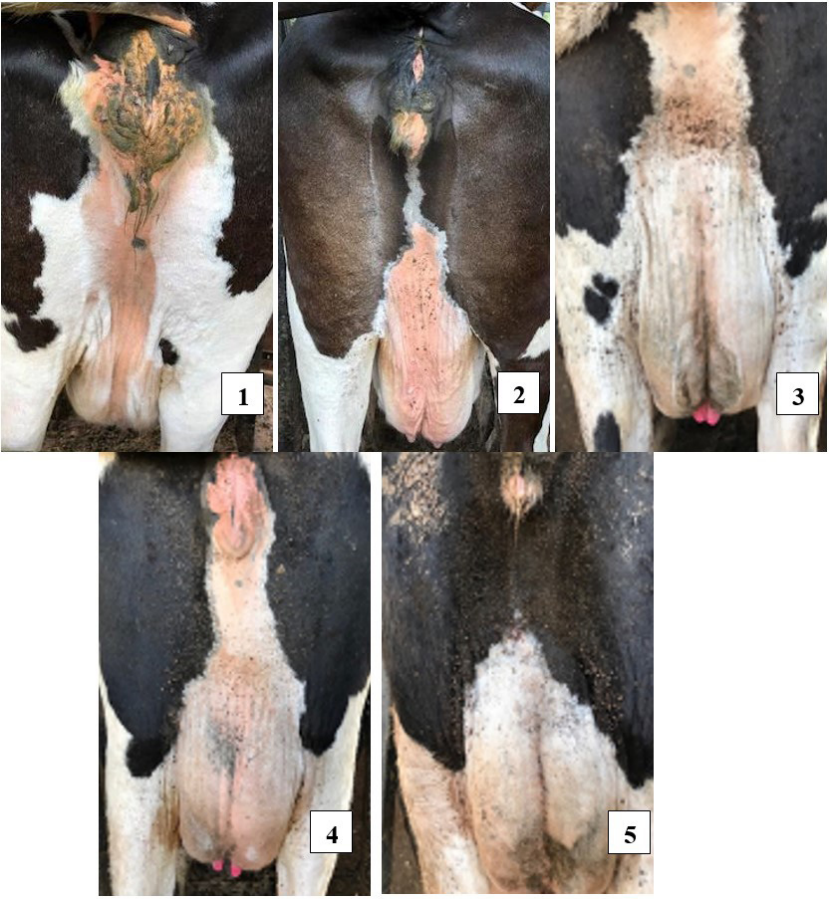
Degree of tick infestation in the perineum, through visual evaluation: 1 – no ticks; 2 - low infestation; 3 - moderate infestation; 4 - high infestation; 5 - very high infestation.

Natural infestation counts of *Rhipicephalus microplus* were obtained through individual inspection of each animal in a cattle crush, at intervals of around 21 days. The tick evaluation was based on the counting methodology of accessing the infestation in the anterior region of one side (head, neck, dewlap, shoulder, thoracic limb and axilla and right side), as proposed by Veríssimo & Oliveira (1994). We also evaluated the degree of tick infestation in the perineum by means of visual evaluation, following the criterion adopted by Mendes (2005), with modification: 1 – no ticks; 2 – low infestation; 3 – moderate infestation; 4 – high infestation; 5 – very high infestation (Figure 1). This was done only to follow and monitor the infestation level, and was not used as a criterion for acaricide application.

### Tick control

Tick control was done using a selective control method for all the animals. Acaricide was only applied when the count on the right anterior third was greater than or equal to 8 ticks measuring 4 mm or more. The control product used was based on 1% flumethrin (Bayticol® pour-on), consisting of 10 ml for each 100 kg of the animal. We chose this acaricide because it could be used on lactating cows, was easy to use, and still controlled the ticks on the farm.

### Body condition assessment

Body condition was assessed visually in terms of the fat and meat coverage of the rump region and the base of the tail, on a scale of points ranging from 1 (very lean) to 5 (very fatty), in units of 0.5 points (Fernandes et al., 2016). This evaluation was started one month after the beginning of the experiment and, thus, a total of 8 evaluations were done, carried out at the same time as the tick evaluations.

### Cost analysis

To analyze the total treatment cost and partial treatment cost, the mean weight of each genetic group was obtained. The price of the acaricide used (Bayticol®) was US$ 22.43 per L. The quantity of product per animal was calculated as 10 ml for every 100 kg. The value of the commercial dollar was R$ 3.79 (U$ 1.00 = R$ 3.79), which was calculated as the average value during the evaluation months and was obtained on January 3, 2020 (IPEA, 2020). The costs of the treatments were calculated based on the average weight of the animals in each genetic group, the quantity of product used, the cost of the product and the number of animals, as shown below:


CTT=Q*C*TA (1)

CTP=Q*C*TR (2)

Where:

CTT = total treatment cost

CTP = partial treatment cost

Q = quantity of product used, in ml

C = cost in US dollars per ml of the product used

TA = total number of animals in the assessment

TR = number of treatments performed

Treatment savings (ET) were obtained from:


ET=CTT−CTP (3)

The cost reduction (RC) was obtained from:


$$RC\;\left(\%\right)=100–\left(CTP*100/CTT\right)$$ (4)

### Statistical analysis

The data on the tick counts were transformed into log_10_ (n+1) to approximate normal distribution of the data. Each variable was analyzed using mixed models. These models included the fixed effects of evaluation, genetic group and interaction evaluation x genetic group, and the animal was used as random effect. The means were compared using the Tukey test (p < 0.05). The percentage of untreated animals were also ascertained. All analyses were performed using the SAS statistical package (SAS, 2002).

## Results

The mean values (log_10_) obtained in each evaluation of the tick count according to genetic group are presented in Table 1. There was a significant difference (p < 0.05) between the genetic groups in each evaluation, and the Holstein breed presented the highest mean infestation levels. These levels differed from the other genetic groups (p < 0.05) in all the evaluations, except in evaluation 1 (Table 1). The infestation level in the Jersey breed was not significantly different (p > 0.05) from that of the Girolando breed in most of the evaluations, and this breed presented the lowest averages (Table 1).

**Table 1:** Average numbers of ticks (log_10_ (n+1)) according to genetic group (H - Holstein; C - crossbreed; J - Jersey; G - Girolando) and standard error in each evaluation.

	**H**	**C**	**J**	**G**
**Jun 2018**	0.43 ± 0.03a	0.37 ± 0.05a	0.28 ± 0.08a	0.22 ± 0.09b
**Jul 2018**	0.41 ± 0.03a	0.24 ± 0.05b	0.19 ± 0.08b	0.24 ± 0.07b
**Aug 2018**	0.47 ± 0.03a	0.32 ± 0.04b	0.14 ± 0.08c	0.20 ± 0.06bc
**Sep 2018**	0.42 ± 0.03a	0.20 ± 0.04b	0.14 ± 0.09b	0.07 ± 0.06b
**Oct 2018**	0.53 ± 0.03a	0.18 ± 0.04b	0.17 ± 0.09b	0.11 ± 0.07b
**Nov 2018**	0.43 ± 0.03a	0.23 ± 0.04b	0.14 ± 0.08b	0.11 ± 0.07b
**Dec 2018**	0.55 ± 0.03a	0.27 ± 0.04b	0.11 ± 0.08b	0.03 ± 0.07b
**Jan 2019**	0.52 ± 0.03a	0.25 ± 0.04b	0.20 ± 0.07b	0.12 ± 0.07b
**Feb 2019**	0.48 ± 0.03a	0.22 ± 0.07b	0.01 ± 0.13b	0.18 ± 0.07b

Averages followed by different lower-case letters in the same line present significant differences (p < 0.05).

Table 2 shows the mean degrees of tick infestation in each breed and according to each evaluation. In June, July and November, the genetic groups did not differ from each other (p > 0.05). In the other months, the Holstein breed presented the highest degree of infestation, which differed from that of the Girolando breed (p < 0.05). In December, the Holstein breed presented higher mean tick infestation than all the other genetic groups (p < 0.05). In general, all the groups differed from each other, and the Holstein breed presented the highest degree of infestation, followed by the crossbreed, Jersey and Girolando groups. There was little or no infestation in the Girolando cows.

**Table 2:** Mean degree of tick infestation and standard error, according to the genetic group (H - Holstein; C - crossbreed; J - Jersey; G - Girolando) in each evaluation.

	**H**	**C**	**J**	**G**
**Jun 2018**	3.2798 ± 0.14a	3.2976 ± 0.22a	3.6709 ± 0.36a	2.6751 ± 0.42a
**Jul 2018**	2.8523 ± 0.13a	2.611 ± 0.22a	2.6773 ± 0.39a	2.1419 ± 0.36a
**Aug 2018**	3.0072 ± 0.13a	2.8087 ± 0.20ab	2.5281 ± 0.36ab	2.3754 ± 0.29b
**Sep 2018**	2.2263 ± 0.13a	2.2421 ± 0.20ab	2.017 ± 0.42ab	1.6067 ± 0.29b
**Oct 2018**	3.1908 ± 0.12a	2.4089 ± 0.20b	2.417 ± 0.42ab	2.1215 ± 0.32b
**Nov 2018**	2.7405 ± 0.13a	2.5313 ± 0.19a	2.2256 ± 0.39a	2.1783 ± 0.31a
**Dec 2018**	3.043 ± 0.13a	2.5767 ± 0.20b	2.1174 ± 0.36b	2.2148 ± 0.30b
**Jan 2019**	2.8183 ± 0.13a	2.8303 ± 0.20ab	2.2435 ± 0.32ab	2.1243 ± 0.34b
**Feb 2019**	4.0018 ± 0.14a	3.7625 ± 0.34a	3.1519 ± 0.64ab	2.384 ± 0.36b

Averages followed by different lower-case letters in the same line present significant differences (p < 0.05).

Table 3 presents the body condition score. This shows that in all the evaluations, the group of animals of the Girolando breed stood out, and the body scores were higher than 3, unlike the Holstein cows, which only exceeded the score of 3 in November.

**Table 3:** Body condition scores of the animals and standard errors according to genetic group (H - Holstein; C - crossbreed; J - Jersey; G - Girolando), in each evaluation.

	**H**	**C**	**J**	**G**
**Jul 2018**	2.70 ± 0.08b	3.13 ± 0.14a	3.18 ± 0.21a	3.39 ± 0.22a
**Aug 2018**	2.86 ± 0.08c	3.31 ± 0.13b	3.24 ± 0.21bc	4.26 ± 0.18a
**Set 2018**	2.97 ± 0.08b	3.16 ± 0.12b	3.12 ± 0.25ab	3.67 ± 0.18a
**Oct 2018**	2.86 ± 0.07b	3.08 ± 0.12b	3.22 ± 0.25ab	3.67 ± 0.20a
**Nov 2018**	3.37 ± 0.08b	3.34 ± 0.12b	3.28 ± 0.24b	3.96 ± 0.19a
**Dec 2018**	2.88 ± 0.08c	3.63 ± 0.12ab	3.22 ± 0.22bc	3.93 ± 0.19a
**Jan 2019**	2.78 ± 0.08b	2.88 ± 0.12b	2.86 ± 0.24ab	3.37 ± 0.21a
**Feb 2019**	2.83 ± 0.08b	3.33 ± 0.19a	3.60 ± 0.37a	3.08 ± 0.22ab

Averages followed by different lower-case letters in the same line present significant differences (p < 0.05).

Table 4 presents data on the animals that were not treated in each genetic group, according to evaluation, followed by the total number of animals evaluated. The Girolando animals had fewest acaricide treatments, and the percentage of untreated animals was higher than 80% in all evaluations. For the Jersey and crossbreed groups, the percentages of untreated animals were higher than or close to 70% in all evaluations, with the exception of the January 2019 assessment for Jersey. Holstein cows, which presented the highest tick infestations, received the greatest numbers of applications of acaricide: the overall average proportion of untreated animals was 34% (minimum 18% and maximum of 50%).

**Table 4:** Percentage of untreated animals in each evaluation, according to the genetic group (H - Holstein; C - crossbreed; J - Jersey; G – Girolando). The total number of animals evaluated in each group is presented in parentheses, and the total number of animals in each evaluation is shown in the last column.

	**H (% (n))**	**C (% (n))**	**J (% (n))**	**G (% (n))**	**Total (n)**
**Jun 2018**	38.78 (49)	68.42 (19)	71.43 (7)	80.01 (5)	80
**Jul 2018**	46.30 (54)	78.95 (19)	83.33 (6)	85.71 (7)	86
**Aug 2018**	38.18 (55)	73.91 (23)	100 (7)	90.91 (11)	96
**Sep 2018**	50 (52)	81.82 (22)	80 (5)	100 (11)	90
**Oct 2018**	18.03 (61)	90.91 (22)	80 (5)	100 (9)	97
**Nov 2018**	45.45 (55)	84.00 (25)	100 (6)	90 (10)	96
**Dec 2018**	18.97 (58)	72.73 (22)	85.71 (7)	100 (10)	97
**Jan 2019**	27.78 (54)	82.61 (23)	55.56 (9)	100 (8)	94
**Feb 2019**	28.26 (46)	71.43 (7)	100 (2)	100 (7)	62
**Average**	**34.3 (484)**	**79.12 (182)**	**81.48 (54)**	**94.87 (78)**	**798**

Table 5 shows the estimated profit obtained by not treating all the animals on the farm, as it had been done before implementation of the selective partial control, considering the quantity of product used for each genetic group according to the average weight of the animals over the total period of nine months of conducting this study.

**Table 5:** Total treatment cost for the herd (TTC), partial treatment cost for the herd (PTC), treatment savings (TS) and cost reduction (CR) in relation to each genetic group (H - Holstein; C - crossbreed; J - Jersey; G – Girolando) (June 2018 to February 2019).

Genetic groups	Total number of evaluations	Numbers of treatments performed	Average weight (kg)	TTC (US$)	PTC (US$)	TS (US$)	CR (%)
H	484	318	590	640.44	420.78	219.65	34.3
C	182	38	469	191.84	40.06	151.79	79.1
J	54	10	386	47.23	8.75	38.49	81.5
G	78	4	509	89.22	4.58	84.64	94.9

Regarding the estimate of the total treatment cost (TTC), it was observed that because the Holstein breed was the predominant breed in the herd and had the highest weight, it presented the highest estimate (US$ 640.44), followed by the crossbreed cows (US$ 191.84), Girolando cows (US$ 89.22) and, lastly, Jersey cows (US$ 47.23). Because the Jersey cows had the lowest weight, they therefore received the lowest dosage of the acaricide product. Regarding the costs of partial treatment (PTC), the expenditure on the Holstein breed was highest (US$ 420.78), followed by that of the crossbreed (US$ 40.06), Jersey (US$ 8.75) and Girolando (US$ 4.58). Regarding the savings made through this treatment method, although on average only 34% of the Holstein cows did not receive any applications, we found that the largest savings (US$ 219.65) were in relation to this breed because it was the genetic group with the highest number of animals in the herd. This group was followed by the crossbreed (US$ 151.79), Girolando (US$ 84.64) and Jersey (US$ 38.49) groups. The reduction in cost represented the reduction in the percentage of TTC for PTC and, thus, the breed that presented the greatest cost reduction was Girolando (94.9%), followed by Jersey (81.5%), crossbreed (79.1%) and Holstein (34.3%).

## Discussion

The results obtained in the present study demonstrated the great susceptibility presented by Holstein animals. This was the breed most affected by ticks and, consequently, it presented the highest frequency of infestations above the tick threshold that had been established for implementation of control. These were therefore the cattle that received the largest number of applications of acaricide. Utech et al. (1978) compared the degree of tick resistance in several cattle breeds and determined that the Holstein breed had the highest sensitivity, and, among breeds of European origin, the Jersey breed had the highest resistance. Veríssimo et al. (2004) found in a Jersey herd that 73% of the tick counts were between 0 and 25 ticks larger than 4.5 mm on one side, thus demonstrating the higher resistance that this breed presents. The same was observed by Teodoro et al. (1994), in comparing the parasite loads of female calves from crossbreed cows that were ½, ^5^/_8_ or ¾ Holstein x *Gir* with those of Holstein, Jersey and Swiss Brown bulls. They found that the calves of the Jersey bulls had fewer ticks than those of the other bulls. In the present study, Jersey animals required fewer applications of acaricide, since they presented low tick counts. The most resistant cows, which received the fewest acaricide applications, were those of the Girolando breed. Lemos et al. (1985) had already shown that ½ and ¼ Holstein x Zebu animals were more resistant to ticks than other degrees of crossbreeding.

In a study on selective tick control in two beef cattle herds, Molento et al. (2013) showed a graph presenting the percentage of tick infestation of each breed that they had studied. From this, it was inferred that those cattle of European origin must have been the ones most frequently exposed to chemicals, because there were greater number of those animals classified as more sensitive to the tick. Signoretti et al. (2006) evaluated selective tick control using chemicals over a two-year period (24 monthly observations) in a crossbreed herd (Holstein x Zebu). These authors found that the treated population comprised, at most, 36% of the herd; in seven non-consecutive evaluations, there was no need to apply acaricide to any animal in the herd, thus demonstrating the efficiency of partial control in that herd. Pereira et al. (2016) successfully spread selective control among dairy farms in the Paraíba valley, state of São Paulo, a region where commercial acaricide products no longer present effectiveness. They reported that on one farm, over a three-month period, it was possible to save on acaricide treatments by 78.9%. The results from immersion tests performed before and one year after starting to use selective partial treatment demonstrated that the acaricide used continued to present efficacy of 100%.

According to Higa et al. (2015), *R. microplus* has been resistant to several chemical classes of ectoparasiticides, thus making it increasingly difficult to control this parasite. Reduction of the number of applications of the product decreases the selection pressure on this parasite from the products currently available on the market. Mendes (2005) demonstrated, through implementing selective control on a farm and monitoring the effectiveness of several acaricide groups, that increased efficacy could be achieved for some products, especially those of the pyrethroid group. If the resistance genes are not fixed in the population and are still in heterozygosis, lower frequency of use of chemicals may give rise to the possibility of reversing forms of resistance that may be linked to dominant genes, thereby maintaining the effectiveness of the product. Therefore, according to Molento et al. (2013), it is important to leave part of the tick population unexposed to the chemical product (refugee population). In the herd of the present study, the flumethrin-based product had been used for four years uninterruptedly, and it continues to be used, even after the end of data collection for this experiment, because partial control of the herd has been implemented as a routine procedure on this farm.

The evaluation of tick infestation according to the degree of perineum infestation, based on the study by Mendes (2005), proved not to be a good tool that could be useful in making decisions about whether or not to treat an animal. Especially among Holstein animals, other regions of the body should be evaluated, especially the anterior third, for decision-making on which animals to treat. Oliveira & Alencar (1990) found higher concentrations of ticks in the posterior part of the animals, which includes the perineum. However, they observed that, in the case of Holstein cattle, there were more ticks in the anterior part of the animals than on the rest of the body. Veríssimo & Oliveira (1994), evaluating crossbreed dairy cattle, observed that there was a high correlation between the total count, made on one side of the body, and the count in the anterior third (head, neck, dewlap, shoulder and forelimb, including the axilla). This showed that it was possible to evaluate the resistance of cattle to ticks by counting only the anterior third of the animal, on one side, and that this may be a criterion for applying the acaricide product. This is a faster methodology for accessing information on tick infestation in susceptible breeds like Holstein, in which there are animals with infestations at levels that easily exceed 100 female ticks parasitizing the whole side. However, with such levels, it would take a long time to evaluate the herd. In our evaluation, we needed to quickly release the animals for them to be able to eat, especially the milking cows, so that there would not be any loss of milk production on the day of the evaluation.

The size of ticks to be considered in the count (4.0 mm) was based on the study by Villares (1941) who successfully evaluated the susceptibility of various breeds of cattle using ticks larger than this criterion (Veríssimo, 2013). According to this infestation criterion, the levels of infestation of the crossbreed, Jersey and Girolando cattle were, on average, between grade 2 (low) and 3 (moderate). Only the Holsteins had a general average of 3 on the infestation scale. Even so, the general average numbers of ticks on the anterior third of the animal in each breed were low (untransformed data): 8.5 for Holstein (minimum average of 5.7 in the July evaluation and maximum of 15.2 in the December evaluation); 1.91 for the crossbreed cattle; 0.96 for Jersey and 0.9 for Girolando. This demonstrates that the tick population was under control, even though the product had not been applied to all the animals of the herd.

Ticks greatly affect weight gain among sensitive animals, in situations of high infestation (O'Kelly & Kennedy, 1981). A body condition score of around 3 is considered ideal in a dairy herd, given that extremes should be avoided, i.e. neither very thin nor very fat animals, so as not to harm milk production and animal health (Fernandes et al., 2016).

The greatest proof of lack of need to use acaricides on tick-resistant animals in the present study was the high body condition score that the Girolando cows presented during all experiment. Almost none of these cows underwent acaricide application over the study period, and yet they had the lowest tick infestations.

Although it was necessary to apply more acaricide to Holstein cattle (reaching 81% of the population in October and December), it was found that it was no longer necessary to apply chemicals at every evaluation, to all animals of this breed of highly susceptible cattle, thus providing savings for the production system. Molento et al. (2013) did not observe any problems arising from selective control in two herds in Rio Grande do Sul and obtained major savings for the production system in the two herds studied. Signoretti et al. (2006) also did not mention any inconvenience relating to selective tick control.

The results obtained from the present study showed that among animals of the Jersey breed and those of European x Zebu crossbreed, especially Girolando, selective control is not only possible, but also recommended as a tick control management method, since the financial benefits are large, without interfering with the performance of the animals. However, care should be taken to evaluate the entire herd at least once a month, and preferably every 21 days. The evaluations intervals were scheduled as 21 days, but this was not always possible to achieve, as this herd is at a government institution where there is no working on weekends and holidays, other than milking and feeding activities, which continued without interruption. We also depended on good weather, with no threat of rain, for the acaricide applications. The average number of days taken for *R. microplus* females to fall off their hosts, recorded in several places around the world, is 22.7 days, as calculated in days from the modal day of female fall presented in Table 5 of Pereira & Labruna (2008). On the 21^st^ day of the interval, few fully engorged females were seen, but after this day had passed, the viewing of teleogynes increased. Thus, we emphasize that the evaluation must be done every three weeks, given that the parasitic phase does not suffer as much from weather conditions due to the fact that the tick is fixed on the host, which maintains a constant body temperature. This differs from the free-living phase, in which the ticks are exposed to environmental conditions (Labruna, 2008). There was no statistical difference in infestation levels in the same breed over the period. From untransformed data, it was noted that the average number of ticks on Holstein cows was almost three times higher in summer (December assessment) than in winter (July assessment). High temperatures and humidity favor the non-parasitic stage of life, thus raising the infestation level of the animals (Labruna, 2008). In the other breeds, this pattern of higher infestations in summer and lower in winter was not observed, such that the lowest infestation level among the crossbreed animals was observed in October, Jersey in February and Girolando in December.

The criterion used for determining whether treatment should be implemented, i.e. presentation of 8 or more ticks larger than 4 mm on the anterior third of one side of the animal, proved to be appropriate, because it kept the tick population under control. According to Brasil (2020), implementation of selective control is advantageous with regard to the cost of treatment of the herd. This relates to lower use of product, reduction of stress caused by parasites and increased presence of parasites that remain susceptible to the active agents available on the market.

From the table of costs presented in this study, it can be seen that for the Girolando, Jersey and crossbred animals, selective control gave rise to reductions in the cost of treatment that were greater than 75%. This can be explained by the inclusion of zebu blood in Girolando and crossbred animals and the greater resistance of Jersey animals to the ticks. This demonstrates that through selective control, the cost of treatment of the herd decreases considerably.

Among Holstein animals, the cost reduction was approximately 35%. Although at first sight this reduction seems to be lower than that of the other breeds, it needs to be borne in mind that this breed had the highest tick infestation levels. Moreover, this breed is one of the largest milk producers in the world and, therefore, this reduction in treatment costs is important. From evaluation of the cost estimates at the present study, selective tick control among the Holstein animals presented the greatest treatment savings (US$ 219.65) because they formed the majority of the herd. When tick control is adopted as a technical measure, it is always perceived by producers as bringing economic and health benefits to the animals on the farm (Amaral et al., 2011; Souza, 2012).

## Conclusion

We concluded that selective tick control is feasible and can be indicated for dairy cattle, even for those that are highly susceptible, such as Holstein cows. In relation to European x Zebu crossbreed and Jersey, this type of control should be recommended as a method of tick control, in view of the benefits that it provides for producers and for those who benefit from their product(s): savings within the production system and less use of chemicals, which will generate fewer residues deposited in the environment, or in the animals or their milk, meat or byproducts, in addition to slowing down the appearance of resistance. This technology also meets the requirements of a population that wants the world to be increasingly free from substances that might be harmful to animal and human health.
